# Research and Simulation Analysis of Life Prediction in Notched Structures of DZ411 Alloy

**DOI:** 10.3390/ma19101938

**Published:** 2026-05-08

**Authors:** Yihui Liu, Wenhao Wang, Xianghua Jiang, Dasheng Wei, Yanrong Wang

**Affiliations:** 1 Beijing Institute of Aeronautical Materials, Aero Engine Corporation of China, Beijing 100095, China; yihui_liu@foxmail.com; 2 School of Energy and Power Engineering, Beihang University, Beijing 100191, China; wang_wen_hao@buaa.edu.cn (W.W.); weidasheng@buaa.edu.cn (D.W.); yrwang@buaa.edu.cn (Y.W.); 3 Jiangxi Research Institute, Beihang University, Nanchang 330096, China

**Keywords:** notch structure, stress relaxation, creep subroutine, creep simulation, endurance life prediction

## Abstract

**Highlights:**

- The “notch strengthening” effect in the DZ411 alloy induced by a ring-notched structure.
- The “notch weakening” effect in the DZ411 alloy induced by a small hole.
- Enhancing the service life of material structures by utilizing the “notch strengthening” effect.
- Avoiding the reduction in the service life of material structures caused by the “notch weakening” effect.

**Abstract:**

In order to investigate the influence of notched structures on creep performance under long-term high-temperature conditions, durability tests were conducted on ring-notched and hole-containing thin tubular specimens of directional columnar-grain DZ411 alloy at 850 °C and 930 °C. The results were compared with those of smooth round rod specimens at same temperatures and stress levels, to evaluate the impact of notched structures described above on the rupture life. Based on the experimental data, a finite element subroutine was developed using a macroscopic phenomenological creep model to simulate the creep deformation behavior of the structural components. The stress relaxation characteristics of the two types of notched structures were analyzed. The results show that the ring-notched structure exhibits significant stress relaxation, leading to a “notch strengthening” effect, which improves the endurance property; conversely, the small-hole structure shows insufficient stress relaxation, resulting in “notch weakening” and a reduction in the endurance property. The developed subroutine demonstrates sufficient engineering accuracy in notch creep simulation. Using creep strain as the fracture criterion, the predicted endurance life showed a deviation from experimental results within the acceptable engineering range, indicating that the subroutine has sufficient engineering accuracy.

## 1. Introduction

Nickel-based superalloys exhibit excellent high-temperature creep resistance and are crucial materials for hot-section components in aerospace and industrial gas turbines [1,2,3]. In turbine blade design, ensuring that the material provides sufficient creep and stress-rupture life is one of the primary criteria for hot-section components [4,5]. Therefore, creep strain is commonly used as the life-limiting parameter for turbine blades [6]. The directionally solidified columnar-grain alloy DZ411 features high strength as well as outstanding oxidation resistance and hot-corrosion resistance. It is used for turbine blades operating below 1000 °C in hot-corrosive environments, as well as for blades in marine and ground-based gas turbines that run long-term under corrosive conditions.

Complex geometric features such as pin fins, staggered ribs, discharge slots, and film-cooling holes are often incorporated into actual turbine blade structures [7,8,9]. These irregular structural shapes and non-uniform load distributions can cause significant stress increases in local regions, thereby inducing stress concentration [10]. At the same temperature, creep strain is greater in high-stress regions and smaller in low-stress regions. As time increases, constraint effects lead to stress redistribution [11], and high-stress regions exhibit stress relaxation behavior.

Previous studies have shown that notch structures have a significant influence on the creep behavior of metallic materials. Guo et al. [12] compared the effect of geometric structure on the fatigue performance of small notched specimens through the high-temperature fatigue test, clarifying the life law and failure mechanism of the two types of notched specimens. Qin et al. [13] investigated the notch weakening phenomenon in the nickel-based single-crystal superalloy DD6 induced by cyclic loading and compared it with the notch strengthening effect observed under static creep conditions. Goyal et al. [14] investigated the notch creep behavior of Cr–Mo ferritic steel. Yu et al. [15] demonstrated a notch-strengthening effect using double-notched plate specimens. Zhang et al. [16,17,18] studied the creep fracture mechanism of nickel-based superalloys, and found that small-hole structures can markedly alter stress distribution and microstructural evolution characteristics, thereby producing either strengthening or weakening effects on creep performance. Nevertheless, research on the creep behavior of directionally solidified DZ411 alloy with different notch features remains relatively scarce. In particular, systematic experimental investigations on the high-temperature creep performance of ring-notched and hole-containing thin tubular specimens are still lacking. This study, based on a creep subroutine, simultaneously investigates both ring notches and small-hole notches. Although these two types of notches share similar arc-shaped geometrical features, they give rise to distinct effects, namely “notch strengthening” and “notch weakening,” respectively. Previous studies have typically focused on a single type of notch effect in isolation, with limited comparative analysis. In contrast, this work provides a direct comparison between these two notch types, thereby elucidating the influence of different structural configurations on material performance. This distinction is primarily attributed to the differences in stress redistribution and constraint effects associated with each notch configuration.

In recent years, scholars have achieved numerous results in creep constitutive modeling and numerical simulation. Wang et al. [19] established a normalized creep model, developed an anisotropic material yield criterion, and realized simulations of creep deformation behavior in real engineering structures as well as long-term life prediction considering stress relaxation. They also implemented a *usermat* user subroutine for the normalized creep model within the general finite-element software ANSYS 19.2 environment, applying it to creep analysis of practical structures such as turbine blades. Mangal et al. [20] developed a micromechanics-based constitutive model for creep in polycrystalline nickel-based superalloys within the Unified Mechanics Theory (UMT) framework. Yang et al. [21] established a viscoplastic constitutive theoretical model that enables rapid and accurate calculation of non-elastic transient stress–strain responses in high-temperature alloys, and they developed a UMAT subroutine for a creep material model in the ABAQUS 2016 environment to perform creep simulations of turbine blades. Although these tools provide strong support for creep simulation and mechanism analysis, their development and validation still rely on comprehensive and systematic experimental data.

This research designed two types of specimens (ring-notched and hole-containing thin tubular specimens) for the DZ411 alloy, and conducted creep rupture tests at 850 °C and 930 °C. The endurance life of the notched structural components was obtained and compared with the results of smooth round rod specimens under the same temperature and stress levels. Meanwhile, based on a macroscopic phenomenological creep model, a creep finite-element subroutine was developed to analyze the stress relaxation characteristics of the two types of notched structures, enabling accurate numerical simulation of the creep behavior of components with different notch geometries.

## 2. Endurance Test of Ring-Notched Specimens

### 2.1. Material Properties of DZ411 Alloy

The elastic properties of the DZ411 alloy at different temperatures are listed in Table 1, and the yield strength values are presented in Table 2.

The alloy has a melting point of 1300 °C and a density of 8344 kg/m^3^. It was fabricated using a vacuum melting and casting process. The standard heat treatment schedule for the castings is as follows:

Solution treatment: 1225 °C ± 10 °C for 2 h, followed by air cooling;

Primary aging: 1120 °C ± 10 °C for 2 h, followed by air cooling;

Secondary aging: 850 °C ± 10 °C for 2 h, followed by air cooling.

### 2.2. Ring-Notched Specimen and Test Procedure

**Figure 1:**
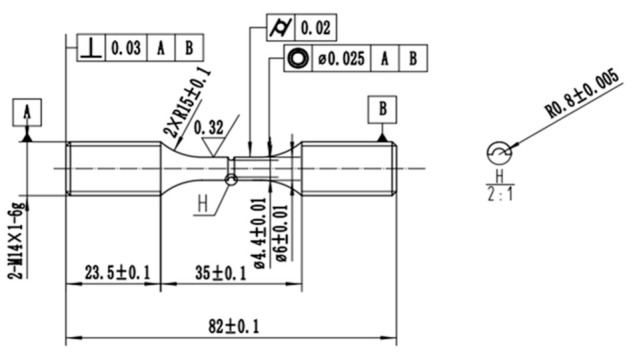
Design drawing of ring-notched specimen (unit: mm).

**Figure 2:**
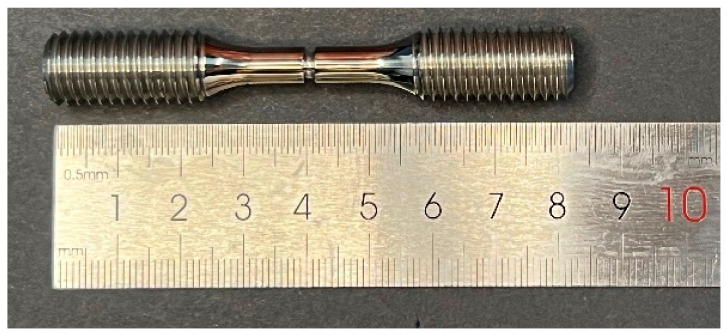
Physical sample of ring-notched specimen.

The structural characteristics frequently present in practical turbine blade configurations, including pin fins and staggered ribs, were idealized and simplified. Accordingly, a ring-notched specimen was designed (Figure 1), and the fabricated specimen is shown in Figure 2.

Creep rupture tests were performed on directionally solidified DZ411 alloy specimens parallel to the solidification direction. The specimens were heated to 850 °C or 930 °C and soaked for 1 h at the target temperature to achieve thermal equilibrium. A preset load was then applied, and the total tensile force was kept constant during testing until failure occurred. The endurance life was subsequently documented.

The creep rupture tests were carried out using a QBR-100 microcomputer-controlled electronic creep/rupture testing machine (Changchun Qianbang, Changchun, China) operated in constant-load control mode. The heating system adopted a three-zone configuration (upper, middle, and lower sections), and each zone was equipped with an S-type thermocouple for precise temperature measurement, ensuring a uniform temperature distribution within the high-temperature furnace and homogeneous heating of the specimen. No extensometer was employed throughout the entire test in order to simplify the testing setup and minimize potential interference.

**Figure 3:**
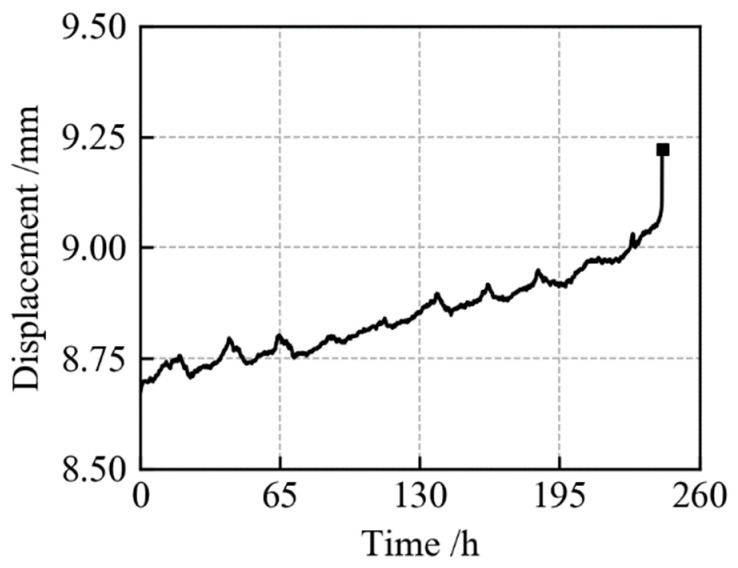
Displacement–time curve of specimen QK-930-320-1.

Taking QK-930-320-1 in Table 3 as an example, its displacement–time curve is shown in Figure 3. However, this displacement represents the overall deformation of the specimen and cannot be used to determine the local strain in the notched region. Therefore, the displacement–time curve can only serve as a supplementary reference and is not suitable for direct comparison with the simulation results.

### 2.3. Endurance Life of Ring-Notched Specimens

The results of the ring-notched creep rupture tests are summarized in Table 3. The specimen identification numbers were assigned according to the following convention: the first term denotes the specimen type; the second term represents the test temperature; the third term indicates the applied load (expressed as the average stress at the minimum cross-section); and the fourth term specifies the sequence number of repeated tests (when no repetition was conducted, only the number “1” is used). A total of two specimens were tested at 850 °C and four specimens at 930 °C, including three repeated tests.

### 2.4. Comparison of Results Between Ring-Notched Specimens and Smooth Specimens

A comparison of the creep endurance lives between the ring-notched specimens and the smooth round rod specimens (fabricated from the same batch of directionally solidified DZ411 alloy) is presented in Table 4. The temperature and loading conditions of the smooth specimens are consistent with those of the notched specimens, and their rupture lives are used for comparison with those of the notched specimens. Under identical temperature and stress conditions, the endurance life of the notched specimens was approximately several to several tens of times longer than that of the smooth specimens.

A multiaxial stress state was established in the local region of the ring-notched structure, where the equivalent stress was lower than the axial tensile stress, thereby suppressing the initiation of microcracks compared with the smooth specimens. Creep deformation of the material induced stress relaxation at the notch root, which further delayed plastic deformation and consequently enhanced the creep endurance life of the specimens.

### 2.5. Dispersion of the Test Results

To evaluate the influence of material scatter on data reliability, repeated creep rupture tests were conducted on ring-notched specimens of directionally solidified DZ411 alloy under the test condition of 930 °C and 320 MPa (see Table 3). The repeated test results indicate that the rupture life under this condition exhibits a certain degree of variation, with a maximum value of 283 h and a minimum value of 243 h. The percentage error was calculated based on the following formula:

(1) $$\mathrm{error}=\frac{t_{\max}-t_{\min}}{\frac{1}{2}\left(t_{\max}+t_{\min}\right)}\times 100\%$$

$t_{\max}$ and $t_{\min}$ denote the maximum and minimum rupture lives, respectively, under the same temperature and stress conditions. The calculated error in the creep endurance life of the notched specimens was approximately 15.2%, which falls within an acceptable range. Considering that experimental data generally exhibit greater scatter under low-stress conditions, it can be inferred that, under other test conditions, the data obtained from a single specimen still possess relatively high representativeness and reliability. Therefore, it is reasonable and scientifically justified to use the results from single-specimen tests as the basis for analysis in most test conditions.

### 2.6. Fracture Morphology of the Ring-Notched Specimens

**Figure 4:**
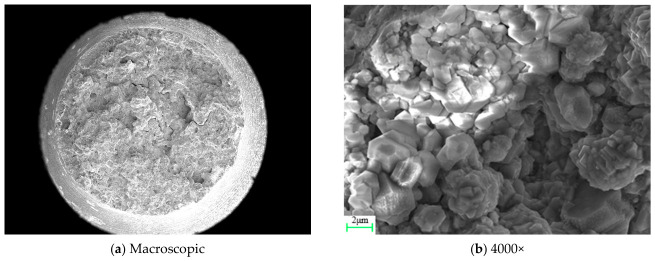
Fracture morphology of specimen QK-850-540-1.

The fracture morphology of specimen QK-850-540-1 is shown in Figure 4. At a magnification of 4000×, the fracture surface exhibits a granular appearance, corresponding to intergranular particle fracture within intergranular failure.

**Figure 5:**
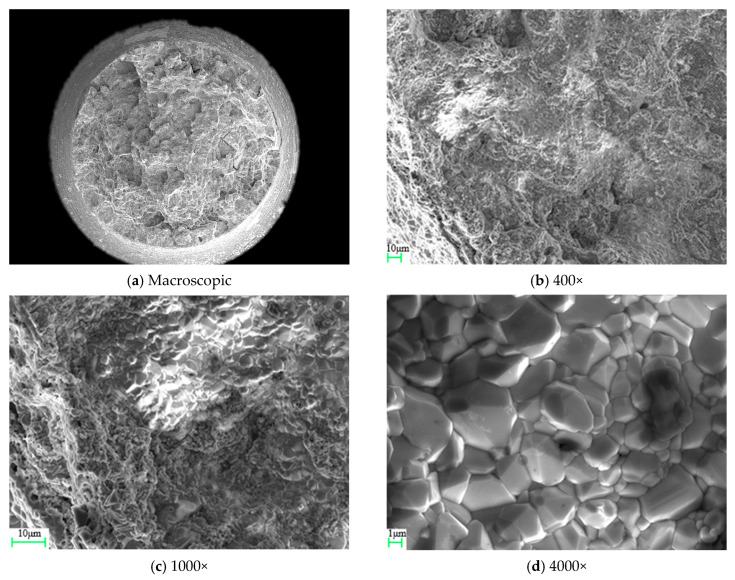
Fracture morphology of specimen QK-930-340-1.

The fracture morphology of specimen QK-930-340-1 is shown in Figure 5. At a magnification of 400×, a “sugar-like” pattern can already be observed, which becomes more pronounced at 1000× and 4000× magnifications. This corresponds to an intergranular fracture morphology characterized by separation along equiaxed grains.

## 3. Endurance Test of Hole-Containing Thin Tubular Specimens

### 3.1. Material Properties of DZ411 Alloy

Material properties are the same as in Section 2.1.

### 3.2. Hole-Containing Thin Tubular Specimen and Test Procedure

**Figure 6:**
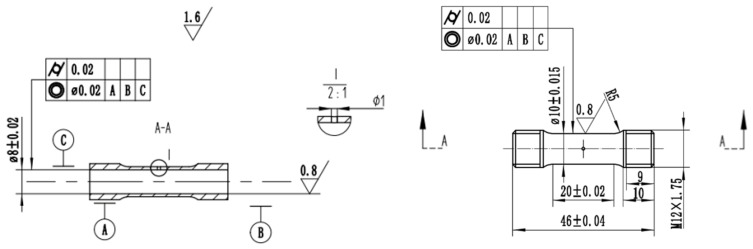
Design drawing of hole-containing thin tubular specimen (unit: mm).

**Figure 7:**
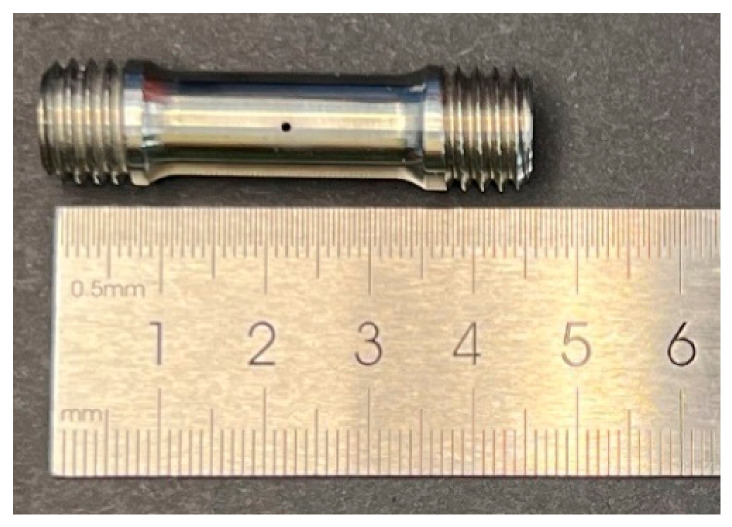
Physical sample of hole-containing thin tubular specimen.

By abstracting and simplifying structural features such as film cooling holes in actual turbine blade structures, a hole-containing thin tubular specimen was designed, as shown in Figure 6. The processed specimen is shown in Figure 7.

Creep rupture tests were performed on directionally solidified DZ411 alloy specimens parallel to the solidification direction. The remaining test procedure and conditions were consistent with those described in Section 2.2.

### 3.3. Endurance Life of Hole-Containing Thin Tubular Specimens

The results of the creep endurance tests for the hole-containing thin tubular specimens are summarized in Table 5. The specimen identification numbering follows the same convention as outlined in Section 2.3, with the test load being the average stress at the minimum cross-sectional area. Additionally, compared with the creep data for smooth round rod specimens obtained in reference [17] under the same temperature and load conditions (see Table 6), the endurance life of the ring-notched specimens in this study was significantly shorter, further confirming the significant impact of notches on endurance life.

### 3.4. Comparison of Results Between Hole-Containing Thin Tubular Specimens and Smooth Specimens

A comparison of the creep endurance lives between the hole-containing thin tubular specimens and the smooth round rod specimens (fabricated from the same batch of directionally solidified DZ411 alloy) is presented in Table 6. The temperature and loading conditions of the smooth specimens are consistent with those of the notched specimens, and their rupture lives are used for comparison with those of the notched specimens. Under identical temperature and stress conditions, the rupture life of the perforated thin tubular specimens was approximately 30% to 71% of that of the smooth specimens.

A multiaxial stress state is formed in the region near the small hole of the hole-containing thin tubular specimen, where the equivalent stress is slightly lower than or close to the tensile stress. The stress relaxation caused by material creep is not significant. The maximum stress at the hole edge is considerably higher than the applied load on the smooth specimen, which accelerates crack propagation and reduces the endurance life of the specimen.

## 4. Creep Model and Subroutine

### 4.1. Creep Model

The model based on normalized parameters expresses the creep strain as:

(2) $$\varepsilon_{c}=\eta_{1}(1-e^{-\eta_{4}\zeta })+\eta_{2}\zeta +\eta_{3}\zeta^{\eta_{5}}$$

In the equation, *t* is time, $\zeta =t/t_{c}$ is the dimensionless time, and $\eta_{i}$ (*i* = 1, 2, 3, 4, 5) is the creep model control parameter with clear physical significance. $t_{c}$ represents the creep endurance life of the material at a given temperature and stress, which can be obtained through corresponding creep rupture tests or endurance life equations for the given temperature and stress, with $0\leq t\leq t_{c}$ and 0≤ζ≤1. Additionally, $\eta_{i}$ (*i* = 1, 2, 3, 4, 5) control the trend of the creep curve, with the conditions $\eta_{i}>0$ and $\eta_{5}>1$.

### 4.2. Creep Subroutine

The creep subroutine utilizes the *usermat* subroutine based on the general finite element software ANSYS. This subroutine is suitable for user-defined material model development and can be used to implement various elastic-plastic material models, including those that account for material damage.

The primary task of the *usermat* subroutine is the definition of the material stress–strain relationship, which includes two aspects: stress updating and the consistent tangent operator matrix. The stress update process calculates the stress increment Δσ from the given strain increment Δε, thereby obtaining the new stress σ. The expression for the consistent tangent operator matrix is given by:

(3) $$D_{\mathrm{ep}}=\frac{\partial \Delta \sigma }{\partial \Delta \varepsilon }$$

There is data exchange between the *usermat* subroutine and the ANSYS main program. Input variables refer to the relevant variables passed from the ANSYS main program to the *usermat* subroutine, while output variables refer to the variables calculated by *usermat* and then passed back to the ANSYS main program. It should be noted that some variables serve as both input and output variables, such as the stress components.

**Figure 8:**
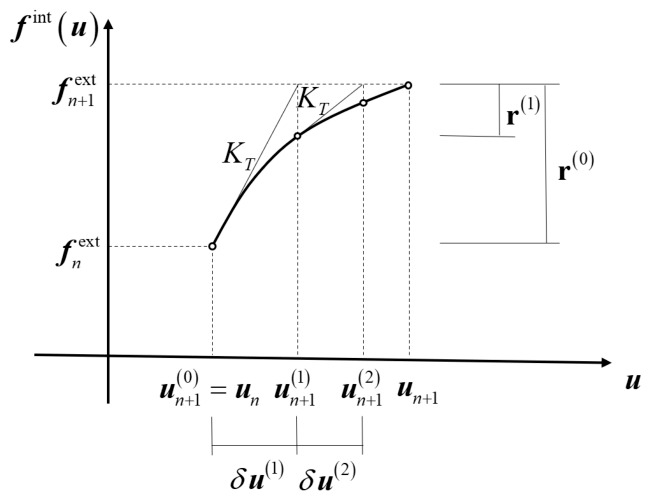
Internal force-displacement iteration process of the incremental finite element equilibrium equation.

The incremental form of the virtual displacement principle is used for programming, with the algorithm being the Newton-Raphson iteration method. The internal force-displacement iteration solution process is shown in Figure 8.

The stress–strain relationship includes the elastic matrix and the elastoplastic matrix, forming a system of equations for the constant stiffness iterative solution. The expression is as follows:

(4) $$d\sigma_{ij}=D_{ijkl,\mathrm{ep}}^{t}\left(d\varepsilon_{kl}-d\varepsilon_{kl}^{T}-d\varepsilon_{kl}^{p}\right)+d\sigma_{ij}^{0}$$

After each iteration, the stress is updated once,

(5) $$\sigma_{\left(k+1\right)}^{t+\Delta t}-\sigma_{0}=D_{\mathrm{ep}}\left(\Delta \varepsilon -\Delta \varepsilon_{T}-\Delta \varepsilon_{c\left(k+1\right)}\right)$$

obtained

(6) $$\begin{array}{c} \left(I+\theta \Delta t\beta_{\left(k\right)}^{t+\theta \Delta t}D_{\mathrm{ep}}C\right)\sigma_{\left(k+1\right)}^{t+\Delta t}\\ \;\;\;\;\;\;\;\;\;\;\;\;\;\;\;\;\;\;\;\;\;\;\;\;\;\;\;\;=D_{\mathrm{ep}}\left(\Delta \varepsilon -\Delta \varepsilon_{T}\right)+\left[I-\left(1-\theta \right)\theta \Delta t\beta_{\left(k\right)}^{t+\theta \Delta t}D_{\mathrm{ep}}C\right]\sigma^{t}+\left(D_{\mathrm{ep}}^{t+\Delta t}-D_{\mathrm{ep}}^{t}\right)\varepsilon_{e}^{t} \end{array}$$

**Figure 9:**
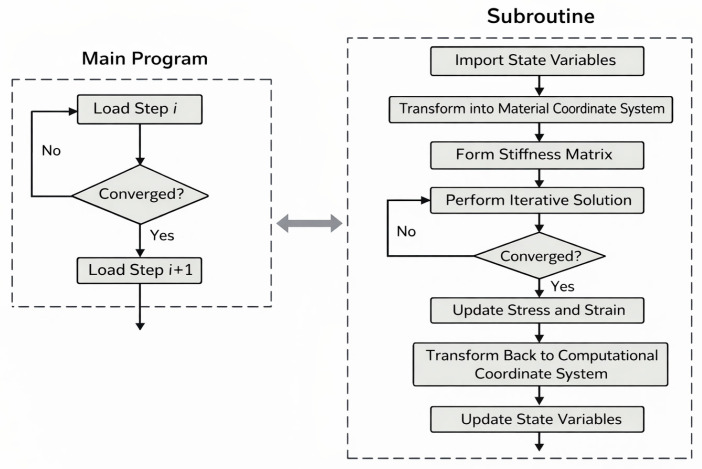
Subroutine call flow.

The above equation requires iteration until the convergence criteria are met. In the equation, $D_{ijkl,\mathrm{ep}}^{t}$ represents the components of the fourth-order tensor consistent tangent operator, and $D_{\mathrm{ep}}$ is the consistent tangent operator matrix. k=0,1,2…, $C=\left[I-\frac{1}{3}mm^{T}\right]$, $m^{T}=\left[1,1,1,0,0,0\right]$, the algorithm is stable when θ is within the value range of [0.5, 1]. The subroutine call flow is shown in Figure 9.

## 5. Creep Deformation Behavior Simulation

The creep deformation behavior of the ring-notched specimens and hole-containing thin tubular specimens is simulated using the creep subroutine presented in Section 4.2.

### 5.1. Creep Behavior Simulation of Ring-Notched Specimens

#### 5.1.1. Model and Mesh of Ring-Notched Specimens

**Figure 10:**
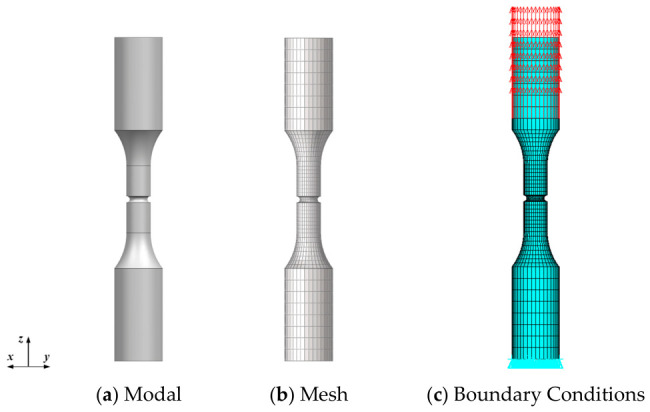
Ring-notched specimen model and boundary conditions.

The specimen model and mesh are established as shown in Figure 10, with 9240 elements and 10,488 nodes. A locally refined mesh is applied in the notch region. The boundary conditions of the specimen are set as shown in Figure 10, where a constraint is applied in the *z*-direction at the bottom of the specimen. Two nodes are selected to apply constraints in the *x* and *y*-directions, and a uniform force load is applied at the top clamped end.

The mesh independence has been verified through a mesh refinement case at the notch root. After 1 h of computation, the errors in the results from different mesh densities were negligible. The creep analysis using the *usermat* subroutine is mesh-independent.

#### 5.1.2. Creep Deformation Behavior Simulation of Ring-Notched Specimens

Using the *usermat* subroutine and considering the anisotropy of DZ411 material, the *z*-direction is taken as the longitudinal direction for the simulation of all test temperatures and stress conditions. Four test cases are set as shown in Table 7, with simulations conducted for six notched specimens.

**Figure 11:**
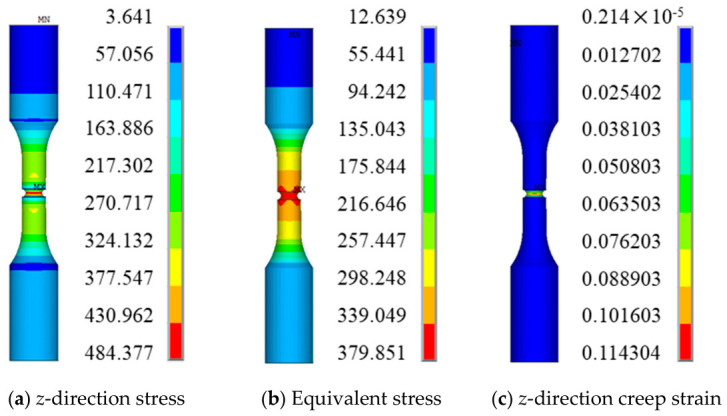
Finite Element Predicted Stress and Strain Distributions (Overall, 394 h).

Since the four simulation cases exhibit similar stress and strain distribution characteristics, case QK-1 is selected as a representative example for presenting and analyzing the numerical results. Case QK-1 corresponds to a creep simulation conducted at 850 °C/540 MPa for 400 h. The distributions of the *z*-direction stress, equivalent stress, and creep strain at the endurance time (i.e., 394 h) are extracted, as shown in Figure 11.

It should be noted that, due to the influence of boundary conditions, the finite element results may exhibit stress values lower than the net-section stress near the boundaries. However, according to Saint-Venant’s principle, the results in the vicinity of the notch are not affected. The subsequent contour plots exhibit the same behavior, and no further explanation will be repeated.

The creep test results for smooth round bar specimens are listed in Table 4. Under the same temperature and net section stress conditions, the endurance life of the smooth round bar specimen is 17.2 h, whereas that of the notched specimen is approximately 23 times longer. Moreover, the creep strain predicted by the present subroutine is significantly higher than that of the corresponding smooth round bar specimen.

**Figure 12:**
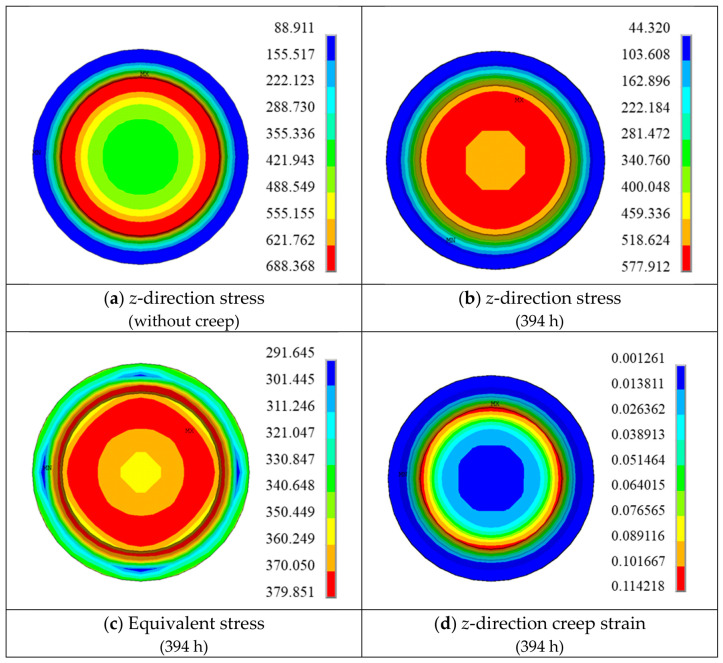
Finite Element Predicted Stress and Strain Results (Notch Root Cross-Section).

To further investigate the local response characteristics, Figure 12 presents the distributions of *z*-direction stress and creep strain across the notched cross-section.

At the rupture time, the maximum *z*-direction stress is not located at the notch root surface, but rather at an interior position near the surface. The variation trend of the equivalent stress is similar to that of the *z*-direction stress; however, its magnitude is significantly lower. In contrast, the maximum *z*-direction creep strain remains located at the notch root.

**Figure 13:**
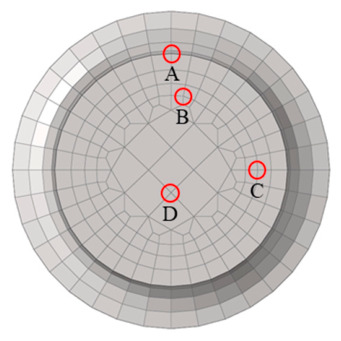
Mesh at the notch root and specific locations of the monitoring points.

**Figure 14:**
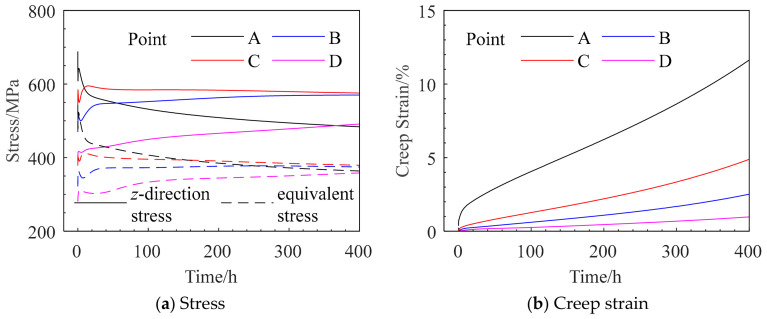
Stress and Creep Strain Curves at the Monitoring Points for Case QK-1.

The selected monitoring points are listed in Table 8, and their specific locations are illustrated in Figure 13. The corresponding stress and creep strain curves are presented in Figure 14.

At the initial stage, the root surface (monitoring point A) exhibits the maximum *z*-direction stress. However, after approximately 5 h, the stress at point A relaxes rapidly and becomes lower than that at point C, indicating that the location of the maximum stress shifts to an interior region near the root surface. The equivalent stress at point A decreases to a lesser extent and falls below that at point C after approximately 146 h. After about 200 h, the equivalent stresses at all four monitoring points approach 400 MPa, and the sectional stress distribution tends to become uniform.

As shown in Figure 14b, the maximum creep strain in the tensile direction occurs at point A, while the minimum value appears at the central point D, demonstrating a non-uniform strain distribution across the section. The internal low-stress, low-deformation region constrains the outer high-stress, high-deformation region, leading to stress redistribution and consequently inducing stress relaxation at and around the notch root.

#### 5.1.3. Fracture Elongation of Ring-Notched Specimens

The maximum creep strain at the endurance time for different simulation cases is extracted and summarized in Table 9. For all cases, the calculated fracture elongation at monitoring point A exceeds 5% and is comparable to the fracture strain of the smooth round bar specimen. This indicates that, for directionally solidified DZ411 alloy, the initiation and propagation of creep cracks are primarily governed by the maximum creep strain. Therefore, the creep strain at monitoring point A can be adopted as the fracture criterion for the notched specimen in the numerical simulation.

Considering that the minimum elongation at fracture of the smooth round bar creep specimens under the same load is 9.85%, a conservative fracture criterion for the notched specimens may be defined as a fracture elongation of 5%, whereas a non-conservative criterion may be taken as 10%. For the four simulation cases, Table 10 presents the corresponding calculated times when the creep strain at monitoring point A reaches 5% and 10%, which are adopted as the predicted rupture lives of the notched specimens by the numerical program.

It can be observed that when the creep strain at evaluation point A reaching 10% is adopted as the fracture criterion, the prediction error of the creep rupture life is within 40% for all cases.

### 5.2. Creep Behavior Simulation of Hole-Containing Thin Tubular Specimens

#### 5.2.1. Model and Mesh of Hole-Containing Thin Tubular Specimens

**Figure 15:**
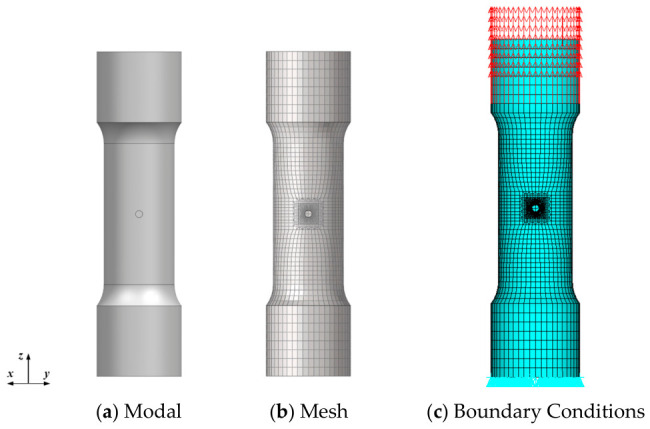
Hole-containing Thin Tubular Specimen Model and Boundary Conditions.

**Figure 16:**
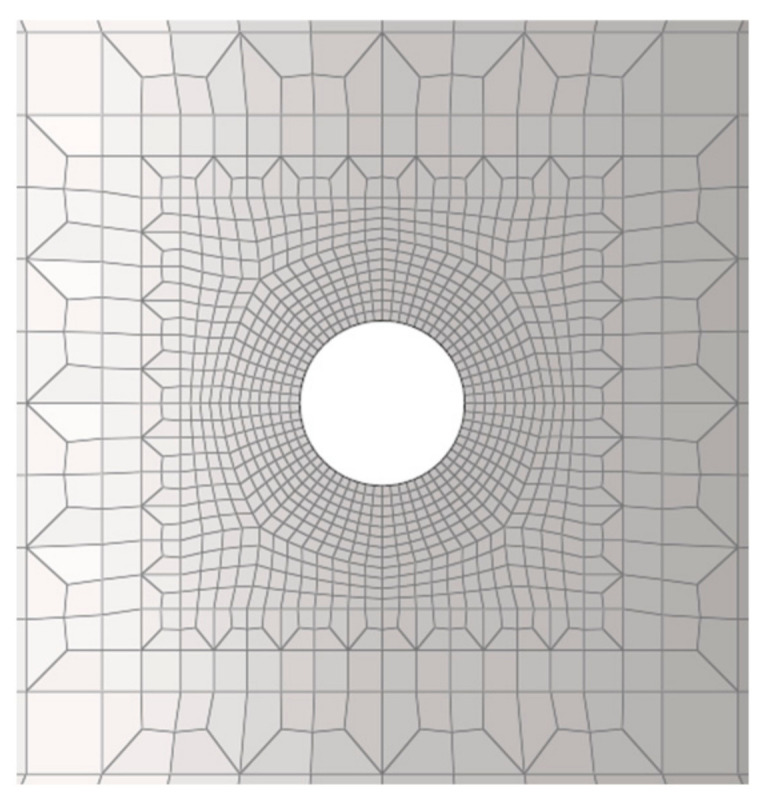
Mesh around the hole of the hole-containing thin tubular specimen.

The specimen model and finite element mesh are shown in Figure 15, comprising 30,656 elements and 35,199 nodes. Local mesh refinement was applied around the hole, as illustrated in Figure 16. The boundary conditions are also presented in Figure 15: the bottom surface of the specimen was constrained in the *z*-direction, two nodes were constrained in the *x*- and *y*-directions to prevent rigid body motion, and a uniformly distributed load was applied at the top gripping end.

Mesh independence was verified by refining the mesh around the small hole. After 1 h of computation, the differences in the results obtained with different mesh densities became negligible. The creep analysis performed using the *usermat* subroutine therefore demonstrates mesh-independence.

#### 5.2.2. Creep Deformation Behavior Simulation of Hole-Containing Thin Tubular Specimens

Using the *usermat* subroutine, the anisotropy of the DZ411 material was taken into account, with the *z*-direction defined as the longitudinal direction. Creep simulations were carried out for the two thin-walled tubular creep specimens listed in Table 11 under different temperature and stress conditions, respectively.

**Figure 17:**
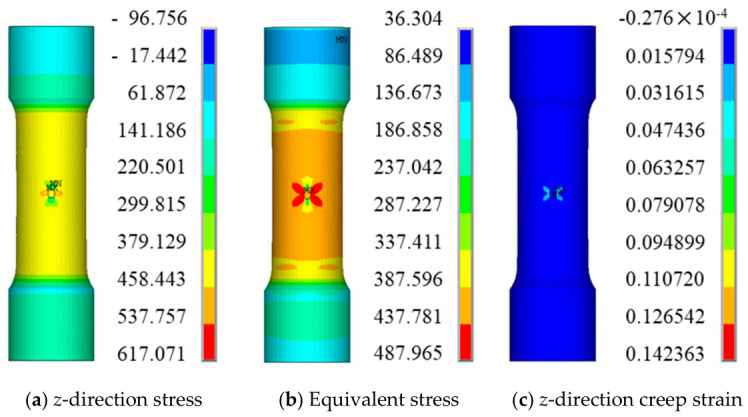
Finite Element Predicted Stress and Strain Distributions (Overall, 67 h).

Since the stress/strain distribution trends of the two cases are similar, Case YG-1 is taken as a representative example for presenting and analyzing the numerical results in this study. The distribution of creep strain is shown in Figure 17. Under the same temperature and net-section stress conditions, the endurance life of the smooth round bar specimen is 226.32 h, whereas that of the hole-containing thin tubular specimen is only about 30% of this value. Moreover, the creep strain level predicted by the subroutine is significantly higher than that of the smooth round bar specimen.

**Figure 18:**
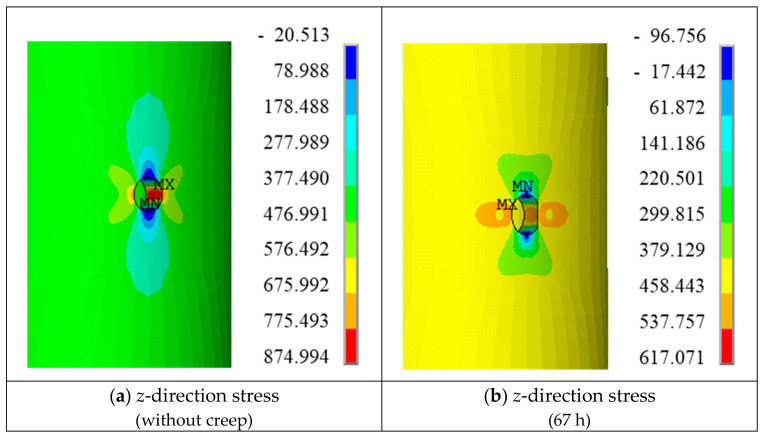

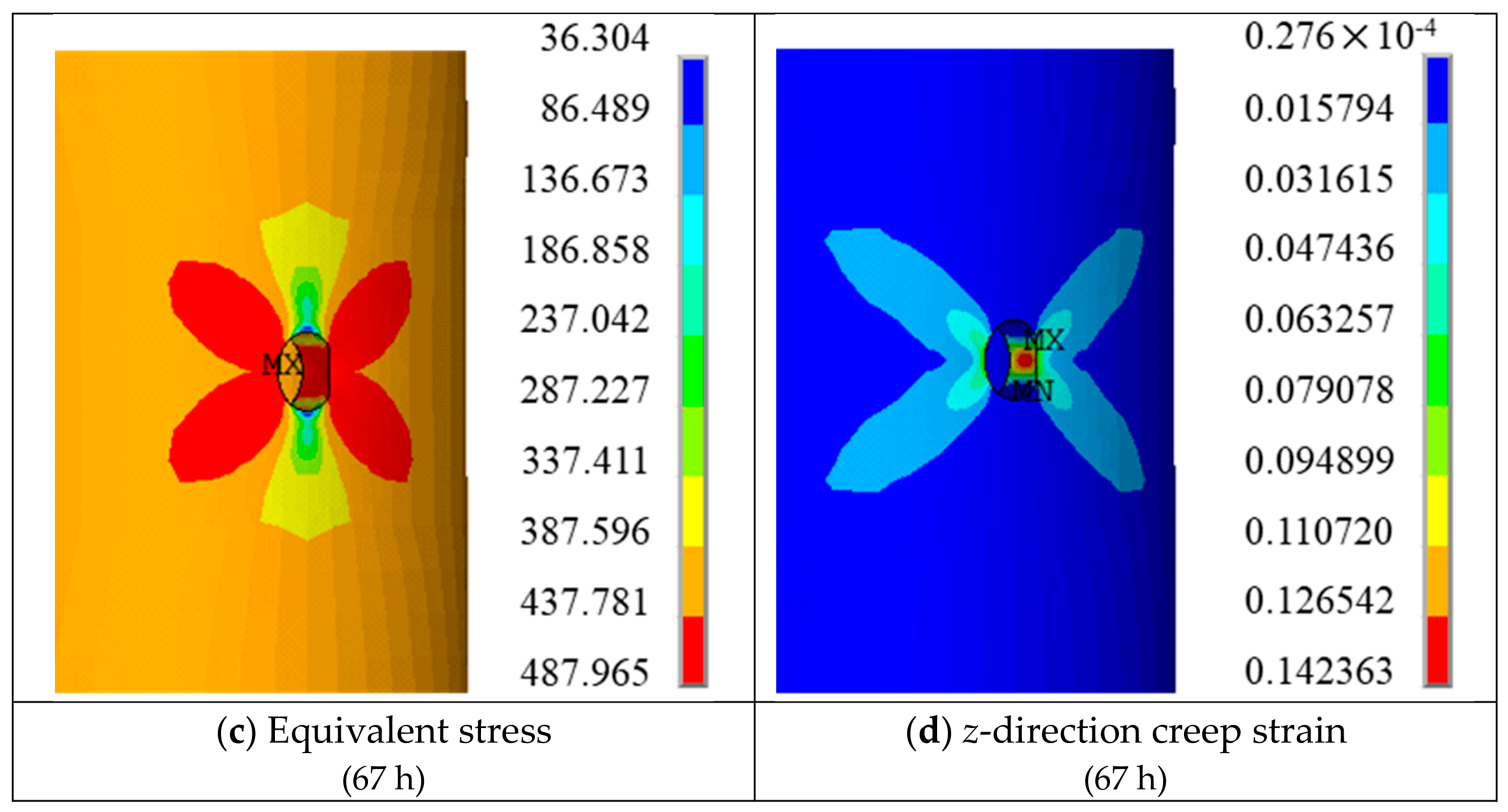
Finite Element Predicted Stress and Strain Results (Hole Edge).

The distributions of the *z*-direction stress and creep strain around the hole are shown in Figure 18. At the rupture time, the maximum *z*-direction stress is not located on the hole surface, but rather at a position inside the hole edge close to the surface. In contrast, the maximum equivalent stress appears near the lower surface beneath the hole.

**Figure 19:**
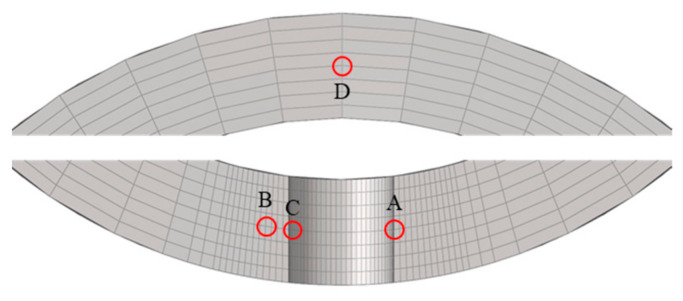
Mesh around the small hole and the specific locations of the monitoring points.

**Figure 20:**
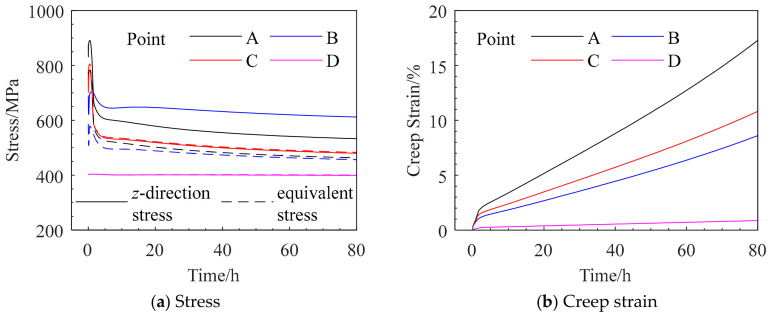
Stress and Creep Strain Curves at the Monitoring Points for Case YG-1.

The selected monitoring points are listed in Table 12, and their specific locations are shown in Figure 19 The corresponding stress and creep strain curves are presented in Figure 20.

The variation trends of the equivalent stress at monitoring points A, B, and C are consistent with those of the *z*-direction stress. However, the equivalent stresses at points A and B are significantly lower than the corresponding *z*-direction stresses, whereas at point C the equivalent stress is slightly higher than the *z*-direction stress. The maximum *z*-direction creep strain is located on the hole surface.

At the initial stage, the hole surface (monitoring point A) exhibits the maximum *z*-direction stress. However, after approximately 1.5 h, the stress at point A relaxes significantly and decreases below that at point B, and the location of the maximum *z*-direction stress shifts to the interior region near the hole surface. The *z*-direction stress and equivalent stress at points A and B decrease by about 320 MPa and 130 MPa, respectively, and the stresses gradually stabilize after 5 h.

It should be noted that the equivalent stresses at the four assessment points have not reached a fully uniform distribution even at rupture, indicating that the stress concentration induced by the small hole cannot be completely relaxed through creep. As shown in Figure 20b, the maximum creep strain in the tensile direction occurs at point A on the hole surface, while the minimum value appears at point D, which is far from the hole. This demonstrates the non-uniform distribution of creep strain in the vicinity of the hole. The constraint imposed by the internal low-stress, low-deformation region on the outer high-stress, high-deformation region leads to stress redistribution, resulting in pronounced stress concentration and a certain degree of stress relaxation at and near the hole surface.

Considering the similarity of the finite element simulation results, the hole-containing thin tubular specimens were simulated only for two temperature/stress combinations and do not cover all experimental temperature/stress conditions; therefore, this part has certain limitations.

#### 5.2.3. Fracture Elongation of Hole-Containing Thin Tubular Specimens

The maximum creep strains corresponding to the endurance time of the specimens in different cases were extracted and are listed in Table 13. The calculated rupture elongations at monitoring point A are approximately 14% and 23%, respectively, which are close to the average rupture elongations of short-term (<80 h and <25 h) round bar creep specimens (13.85% and 15.58%).

Assuming that the initiation and propagation of creep cracks in the columnar-grained DZ411 material are controlled by the maximum creep strain, the creep strain at monitoring point A is adopted as the fracture criterion for the notched specimens in the numerical analysis. Alternatively, if the initiation and propagation of creep cracks are assumed to be governed by the maximum equivalent stress, the creep strain at monitoring point C is taken as the fracture criterion for the notched specimens in the numerical analysis.

For a conservative assessment, the rupture elongation of the hole-containing thin tubular specimen may be taken as 10%. Considering the stress concentration in the hole-containing thin tubular specimen and that the rupture elongation of some short-term creep specimens can exceed 15%, a value of 15% may be adopted for the non-conservative case.

It should be noted that, for DZ411 material, a shorter creep rupture life of the standard specimens corresponds to a higher fracture elongation. Accordingly, when defining the fracture criteria for the two types of specimens, different values were selected based on their respective rupture lives.

The ring-notched specimens exhibit relatively long rupture lives; the corresponding average fracture elongation of the standard specimens at comparable lifetimes is approximately 8.10%, and thus a value of 10% was adopted. In contrast, the hole-containing thin-walled tubular specimens show shorter rupture lives; the corresponding average fracture elongation is about 14.54%, and therefore 15% was selected as the fracture criterion.

For the two simulation cases, the calculation times corresponding to creep strains of 10% and 15% at assessment points A and C were extracted, respectively, as listed in Table 14. According to engineering application requirements, the specific location of the monitoring point and its allowable creep strain can be determined in the program. Combined with the simulation results, the endurance life of the hole-containing thin tubular specimen can then be obtained.

It can be observed that when the creep strain at monitoring point A reaches 15% and is adopted as the fracture criterion, the prediction error of the endurance life is within 30% for all cases.

### 5.3. Analysis and Discussion of Creep Simulation and Experimental Scatter

#### 5.3.1. Comparison of Stress Relaxation Effects

The stresses of both types of notches exhibit significant relaxation over time. Monitoring point A corresponds to the location with the maximum initial stress. The equivalent stresses at point A at different times were extracted from Figure 14 and Figure 20, and the stress relaxation effects were subsequently calculated, as summarized in Table 15. Considering that, at the initial stage of the test, the local notch region undergoes pronounced stress redistribution, the equivalent stress was extracted starting from 5 h onward.

As shown in Table 15, with the continuous progression of creep, the stress at the notch root of the ring-notched specimen undergoes sustained relaxation until fracture. In contrast, the hole-containing thin tubular specimen completes most of its stress relaxation within approximately 5 h, and subsequent creep does not lead to further significant stress relaxation.

Furthermore, the equivalent stress values at four monitoring points for both types of notched specimens at the end of the calculation were extracted, as listed in Table 16. $S_{\max}$ is the maximum stress value among the four monitoring points, while $S_{\min}$ is the minimum stress value among the four monitoring points. The uniformity of the equivalent stress distribution was evaluated as $\left(S_{\max}-S_{\min}\right)/\frac{1}{2}\left(S_{\max}+S_{\min}\right)\times 100\%$. The value is 5.7% for the ring-notched specimen and 18.7% for the hole-containing thin tubular specimen, indicating that the latter exhibits a significantly higher degree of equivalent stress distribution uniformity than the former.

#### 5.3.2. Prediction Error Analysis

When comparing the model predictions with the experimental results, the uncertainties associated with both the material and the experimental procedures should be taken into account. In practice, experimental data commonly exhibit a certain degree of scatter, which can mainly be attributed to two factors.

First, variations in the casting process may introduce microstructural defects during solidification, such as shrinkage cavities, porosity, and segregation, leading to fluctuations in the mechanical properties among different specimens.

Second, microstructural inhomogeneity may also contribute to the observed variability. Local segregation of alloying elements and differences in grain orientation can significantly affect the high-temperature creep behavior, resulting in variations in performance even among specimens from the same batch.

On this basis, the predictions obtained from numerical simulations can be regarded as an idealized estimation of the average material response. Therefore, deviations between the predicted results and the actual endurance life of individual specimens are reasonable and unavoidable. In this context, discrepancies within 40% are generally considered to fall within an acceptable engineering range.

These results indicate that the adopted model exhibits a reasonable level of accuracy and provides meaningful engineering reference for predicting the creep life of high-temperature alloys. Further improvement in the predictive capability of the model, however, requires additional and more comprehensive experimental data to calibrate and refine the model parameters.

## 6. Conclusions

(1) Under the same temperature and net-section stress conditions, the rupture life of the ring-notched specimen is significantly higher than that of the smooth bar creep test, exhibiting a “notch strengthening” effect. In contrast, the rupture life of the hole-containing thin tubular specimen is much lower than that of the smooth bar creep test under the same temperature and stress conditions, exhibiting a “notch weakening” effect.
(2) The developed creep subroutine demonstrates strong engineering applicability and can effectively reproduce the “notch strengthening” and “notch weakening” phenomena observed in directionally solidified materials. When the creep strain at the monitoring point reaches 10% (for ring-notched specimens) and 15% (for specimens with a small hole), respectively, as the fracture criterion, engineeringly acceptable predictions of endurance life can be obtained.
(3) All tests conducted in this study did not employ extensometers for creep strain measurement; therefore, direct comparison between experimental and simulated data is limited due to the lack of experimental measurements. In future work, techniques such as DIC may be considered to supplement creep strain measurements in the local notch region.

## Figures and Tables

**Table 1:** Elastic properties of DZ411 alloy at different temperatures.

*T*/°C	25	100	200	300	400	500	600	700	800	900	1000	1100
*E*/GPa	130	128	126	123	118	114	110	106	101	95	86	80
*G*/GPa	60.01	57.49	54.98	52.91	50.62	48.91	47.06	44.55	41.08	37.99	34.48	33.00
*ν*	0.361	0.363	0.365	0.368	0.372	0.376	0.381	0.385	0.389	0.407	0.418	0.430

**Table 2:** Yield strength of DZ411 alloy at different temperatures.

*T*/°C	20	650	700	800	900	980	1000
Vertical Orientation *σ*_0.2_/MPa	1035	790	915	730	465	345	260
Horizontal Orientation *σ*_0.2_/MPa	910	760	790	850	510	-	280

**Table 3:** Summary of creep tests on ring-notched specimens.

Serial Number	Identification Numbers	Temperature/ °C	Stress/MPa	Endurance Life/h
1	QK-850-540-1	850	540	393.80
2	QK-850-510-1	850	510	513.83
3	QK-930-340-1	930	340	215.33
4	QK-930-320-1	930	320	242.54
5	QK-930-320-2	930	320	283.00
6	QK-930-320-3	930	320	242.57

**Table 4:** Comparison of endurance life between ring-notched specimens and smooth specimens.

Temperature/°C	Stress/MPa	Endurance Life/h		Life Ratio (Notched/Smooth)
		Smooth Specimen	Notched Specimen	
850	540	17.20	398.80	23.19
850	510	36.94	513.83	13.91
930	340	21.62	215.33	9.96
930	320	37.97 28.07	242.54 283.00 242.57	7.75

**Table 5:** Summary of creep tests on hole-containing thin tubular specimens.

Serial Number	Identification Numbers	Temperature/°C	Stress/MPa	Endurance Life/h
1	YG-850-420-1	850	420	67.08
2	YG-850-420-2	850	420	68.61
3	YG-850-450-1	850	450	58.46
4	YG-930-290-1	930	290	56.91
5	YG-930-320-1	930	320	18.84
6	YG-930-320-2	930	320	14.83
7	YG-930-320-3	930	320	22.11

**Table 6:** Comparison of endurance life between hole-containing thin tubular specimens and smooth specimens.

Temperature/°C	Stress/MPa	Endurance Life/h		Life Ratio (Notched/Smooth)
		Smooth Specimen	Notched Specimen	
850	420	226.32	67.08 68.61	0.30 0.30
850	450	128.04	58.46	0.46
930	290	57.30	40.65	0.71
930	320	37.97 28.07	18.84 14.43 22.11	0.57 0.44 0.67

**Table 7:** Summary of Creep Simulation Cases for Ring-Notched Specimens.

Case	Temperature/°C	Stress/MPa	Computation Time/h	Endurance Time/h
QK-1	850	540	400	393.80
QK-2	850	510	600	513.83
QK-3	930	340	250	215.33
QK-4	930	320	300	242.54 283.00 242.57

**Table 8:** Monitoring Points for Ring-Notched Specimens.

Monitoring Point	Time/h	Location
A	0	Point of maximum *z*-direction stress
	394	Point of maximum creep strain
B	394	Point of maximum *z*-direction stress
C	394	Point of maximum equivalent stress
D	-	Center point of the notched cross-section

**Table 9:** Calculated Creep Strain of Ring-Notched Specimens under Different Simulation Cases.

Specimen	Endurance Life/h	Computation Time/h	Calculated Creep Strain (Monitoring Point A)/%	Fracture Elongation (Smooth Specimen)/%
QK-850-540-1	393.80	394	11.43	14.56
QK-850-510-1	513.83	514	8.60	15.17
QK-930-340-1	215.33	215	9.83	14.77
QK-930-320-1	242.54	243	6.96	9.85 10.56 9.85
QK-930-320-2	283.00	283	8.21	
QK-930-320-3	242.57	243	6.96	

**Table 10:** Computational time for different simulation cases of the ring-notched specimens.

Case	Temperature/°C	Stress/MPa	Creep Strain/%	Computation Time/h	Creep Strain/%	Computation Time/h
QK-1	850	540	5.00	144	10.0	349
QK-2	850	510	5.00	258	10.0	596
QK-3	930	340	5.00	108	10.0	218
QK-4	930	320	5.00	170	10.0	333

**Table 11:** Summary of Creep Simulation Cases for Hole-Containing Thin Tubular Specimens.

Case	Temperature/°C	Stress/MPa	Computation Time/h	Endurance Time/h
YG-1	850	420	80	67.08 68.61
YG-2	930	320	20	18.84 14.43 22.11

**Table 12:** Monitoring Points for hole-containing thin tubular specimens.

Monitoring Point	Time/h	Location
A	0	Point of maximum *z*-direction stress
	67	Point of maximum creep strain
B	67	Point of maximum *z*-direction stress
C	67	Point of maximum equivalent stress
D	67	Central point away from the hole

**Table 13:** Calculated creep strain of hole-containing thin tubular specimens under different simulation cases.

Case	Endurance Life/h	Computation Time/h	Computation Creep Strain/%	
			Monitoring Point A	Monitoring Point C
YG-1	67.08	67	14.24	9.00
YG-2	18.84	19	23.39	14.66

**Table 14:** Computation time of hole-containing thin tubular specimens under different simulation cases.

Case	Temperature/°C	Stress/MPa	Monitoring Point	Creep Strain/%	Computation Time/h
YG-1	850	420	A	10.00	47
				15.00	71
			C	10.00	75
				15.00	105
YG-2	930	320	A	10.00	10
				15.00	14
			C	10.00	15
				15.00	20

**Table 15:** Comparison of stress relaxation magnitude between two types of notched specimens.

Stress Relaxation Effect/%	5 h	10 h	20 h	40 h	60 h	80 h	100 h	200 h	300 h	400 h
Ring-Notched Specimen	11.36	16.81	19.64	21.31	22.81	24.17	25.35	29.36	31.73	33.28
Hole-Containing Thin Tubular Specimen	32.86	33.94	35.88	38.38	39.87	40.86				

**Table 16:** Equivalent stress uniformity of two types of notched specimens.

Monitoring Point	A	B	C	D
Ring-Notched Specimen, 400 h	363.63 MPa	374.59 MPa	379.21 MPa	358.31 MPa
Hole-Containing Thin Tubular Specimen, 80 h	463.13 MPa	456.55 MPa	483.17 MPa	400.63 MPa

## Data Availability

The original contributions presented in this study are included in the article. Further inquiries can be directed to the corresponding author.

## Abbreviations

The following abbreviations are used in this manuscript:

$\sigma_{ij}/\mathrm{MPa}$	Stress component
σ	Stress
$\varepsilon_{ij}/\%$	Strain component
ε	Strain
$\varepsilon_{c}/\%$	Creep strain
$\varepsilon_{r}/\%$	Creep fracture elongation
$D_{ijkl,\mathrm{ep}}^{t}$	Components of the fourth-order tensor consistent tangent operator
$D_{\mathrm{ep}}$	Consistent tangent operator matrix
I	Unit vector
ζ	Normalized creep time
$\eta_{1}$,$\eta_{2}$,$\eta_{3}$,$\eta_{4}$,$\eta_{5}$	Parameters in creep model
$t_{c}/h$	Creep rupture time
error	Iteration error
